# Resting-State Oscillatory Activity in Children Born Small for Gestational Age: An MEG Study

**DOI:** 10.3389/fnhum.2013.00600

**Published:** 2013-09-24

**Authors:** Maria Boersma, Henrica M. A. de Bie, Kim J. Oostrom, Bob W. van Dijk, Arjan Hillebrand, Bernadette C. M. van Wijk, Henriëtte A. Delemarre-van de Waal, Cornelis J. Stam

**Affiliations:** ^1^Department of Clinical Neurophysiology, VU University Medical Center, Amsterdam, Netherlands; ^2^Neuroscience Campus Amsterdam, Amsterdam, Netherlands; ^3^Department of Pediatrics, VU University Medical Center, Amsterdam, Netherlands; ^4^Department of Pediatric Psychology, VU University Medical Center, Amsterdam, Netherlands; ^5^Department of Physics and Medical Technology, VU University Medical Center, Amsterdam, Netherlands; ^6^Research Institute MOVE, VU University Amsterdam, Amsterdam, Netherlands; ^7^Department of Pediatrics, Leiden University Medical Center, Leiden, Netherlands

**Keywords:** magnetoencephalography, spectral power, children, small for gestational age, SGA, IQ

## Abstract

Growth restriction *in utero* during a period that is critical for normal growth of the brain, has previously been associated with deviations in cognitive abilities and brain anatomical and functional changes. We measured magnetoencephalography (MEG) in 4- to 7-year-old children to test if children born small for gestational age (SGA) show deviations in resting-state brain oscillatory activity. Children born SGA with postnatally spontaneous catch-up growth [SGA+; six boys, seven girls; mean age 6.3 year (SD = 0.9)] and children born appropriate for gestational age [AGA; seven boys, three girls; mean age 6.0 year (SD = 1.2)] participated in a resting-state MEG study. We calculated absolute and relative power spectra and used non-parametric statistics to test for group differences. SGA+ and AGA born children showed no significant differences in absolute and relative power except for reduced absolute gamma band power in SGA children. At the time of MEG investigation, SGA+ children showed significantly lower head circumference (HC) and a trend toward lower IQ, however there was no association of HC or IQ with absolute or relative power. Except for reduced absolute gamma band power, our findings suggest normal brain activity patterns at school age in a group of children born SGA in which spontaneous catch-up growth of bodily length after birth occurred. Although previous findings suggest that being born SGA alters brain oscillatory activity early in neonatal life, we show that these neonatal alterations do not persist at early school age when spontaneous postnatal catch-up growth occurs after birth.

## Introduction

Of all live-born neonates, a small percentage is born small for gestational age (SGA). This percentage depends on the definition used and ranges between 2 and 10%. SGA born children are characterized by a decreased body weight and/or length and diminished head circumference at birth. Children born SGA suffered from suboptimal intra-uterine conditions that lead to underdevelopment of the body and the brain (de Bie et al., 2010; Frisk et al., 2002; Mallard et al., 2000; Rehn et al., 2004; Saenger et al., 2007; Toft et al., 1995; Tolsa et al., 2004). The most common cause of intra-uterine growth restriction (IUGR) is placental insufficiency (Mallard et al., 2000; Rehn et al., 2004). Other factors that influence fetal growth are maternal age and nutrition, placental function and size, smoking, genetic factors, endocrine factors, and sex of fetus. The majority of SGA born children show catch-up growth in the first 2 years of life (SGA+), and approximately 10% lack catch-up growth (SGA−), showing persistent short stature (Saenger et al., 2007). SGA inclusion criteria and definitions are described in the methods section. Besides physical dysregulation, SGA is associated with decreased levels of intelligence and cognitive abilities in children and adults (de Bie et al., 2010; Lundgren et al., 2001; Strauss, 2000). Interestingly, catch-up growth during the first years of life is associated with relatively better cognitive outcome (Frisk et al., 2002; Lundgren et al., 2001; Saenger et al., 2007).

The period during which SGA born children suffer from intra-uterine growth restriction is generally unknown since frequent monitoring of fetal growth with ultrasound echo is not a common procedure. Dependent on the timing of growth restriction, several brain maturation processes can be affected. In the last trimester of pregnancy, proliferation, and migration of neurons is being completed while maturational processes such as synaptogenesis, dendritic aborization, and myelination start to connect the neurons (Dubois et al., 2008; Ment et al., 2009; Rees et al., 2008; Tolsa et al., 2004; Volpe, 2000). Growth restriction might affect these basic maturation processes and can have long-term consequences.

A few studies have investigated the effects of being born SGA on brain anatomy later in life with magnetic resonance imaging (MRI). Martinussen and colleagues reported on lower total brain volume with reduced white matter volume but no significant differences in gray matter volume for term born SGA 15-year-old adolescents when compared to healthy adolescents (Martinussen et al., 2005, 2009). These results are in line with the results of our recent MRI study in children born SGA at early school age (4- to 7-year-old) (de Bie et al., 2011). This study further differentiated between SGA children with catch-up growth (SGA+) and without catch-up growth (SGA−). Children born SGA demonstrated smaller brains with lower white matter volumes and a smaller cortical surface area. SGA+ children constituted an intermediate between children born appropriate for gestational age (AGA) and SGA− children with respect to these brain parameters with a linear trend ordered from highest volumes and surface area in AGA to SGA− via SGA+ children. Furthermore, both SGA subgroups showed regional differences in cortical thickness most pronounced in the anterior and inferior prefrontal cortex, with SGA− children having the thickest cortex, to intermediate in SGA+ children and lowest in AGA.

Brain oscillatory activity can be recorded with electro- and magnetoencephalography (EEG/MEG). These oscillations are assumed to originate from large neuronal networks that synchronize their activity in the brain areas underlying the sensors. The amplitude of the recorded signals depends on the number of neurons firing in synchrony, which in turn depends on the local connectivity patterns between excitatory and inhibitory neurons as well as on local synaptic density. Development of this oscillatory activity in healthy children is characterized by increases in the amplitude of high frequency oscillations (alpha, beta, and gamma bands) and a reduction in the amplitude of slow oscillations (delta and theta bands) (Okumura et al., 2006; Uhlhaas et al., 2009; Gmehlin et al., 2011). As brain anatomy is affected in SGA children, the development of normal oscillatory activity patterns might also be affected. In an earlier study investigating the effect of being born SGA on brain oscillatory activity, Ozdemir et al. (2009) recorded the EEG of 40 term SGA, and 20 term AGA infants in their first week after birth, and in their first and third month, all during sleep. The authors reported that in all records the amplitude levels were significantly lower for the SGA group than for the AGA group. Furthermore, SGA showed higher relative power for low frequencies and less power for higher frequencies. The authors interpreted their findings as a delay in electrophysiological maturation in term SGA infants.

An advantage of MEG over EEG is that it is a more child-friendly recording technique as the helmet with inbuilt sensors replaces the need to glue electrodes on the head which could be distressing for children. Moreover, the MEG scanner has a 151-sensor array resulting in higher spatial resolution and it is insensitive to the effects of skull-thickness and skin and scalp conductivity, which might have biased the results of Ozdemir and colleagues. In the present study, we used MEG to record brain oscillations during an eyes-closed resting-state condition in SGA and AGA born children at early school age (4–7 years old). The aim of our study was to investigate whether and how oscillatory brain activity is affected in 4- to 7-year-old term born SGA born children in which postnatal catch-up growth has occurred. To answer this question, we calculated the absolute and relative power spectra for several frequency bands and tested for differences between children born SGA and AGA.

## Materials and Methods

### Subjects

This study presents baseline data from a longitudinal project that studies the effects of growth hormone (GH) therapy on brain development and cognition in children born SGA (Dutch Trial Register: NTR 865). The complete project included neuropsychological assessment as well as MRI and MEG investigation and was performed at the VU University Medical Center from March 2007 until April 2010.

Inclusion period of children for MEG scanning ran from March 2008 till October 2009, after which the MEG was replaced by a new system. The study was approved by the ethics committee of the VU University Medical Center. Written informed consent was obtained from the parents or guardians of each child and obtained according to the Declaration of Helsinki (BMJ, 1991; Vol. 302: p. 1194).

Exclusion criteria were: (1) severe prematurity below 34 weeks, (2) complicated neonatal period with signs of severe asphyxia, defined as an Apgar score <7 after 5 min., (3) multiple birth, (4) growth failure caused by somatic or chromosomal disorders or syndromes (except for Silver–Russell syndrome), (5) previous or present use of medication that could interfere with growth or GH treatment, and (6) severe learning disability (IQ < 70). Additional exclusion criteria based on the MEG recordings are described in the data processing section below.

Both SGA and AGA groups were in the age range of 4–7 years old at the moment of the MEG recordings. We included 12 AGA born children and 18 children that were born SGA. Of the 18 SGA children, 14 showed postnatal catch-up growth (SGA+). Following the International Small for Gestational Age Advisory Consensus Board Development Conference Statement (Lee et al., 2003), SGA was defined by a birth weight and/or length less than or equal to −2 SD, adjusted for gender and gestational age; SGA+ was defined as postnatal catch-up growth with an actual height of less than 2 SD below the Dutch population reference mean; and SGA− as persistent postnatal growth failure based on an actual height of less than 2.5 SD below this mean (Fredriks et al., 2000). SGA− children were not included in the current study. AGA was defined as birth weight and length above −2 SD, without known history of prenatal growth restriction. SGA children were selected from the pediatric hospitals in The Netherlands.

Three children (two AGA, one SGA+) were excluded from further processing since they showed minor abnormalities in their MEG recordings (see description in Data Processing); 10 AGA born and 13 SGA+ children were finally included in this study.

### Magnetoencephalography recordings

Since all children included in our study underwent an MRI dummy scanner training session (de Bie et al., 2010), we used this session to shortly explain the MEG scanning procedure as well. We showed and fitted the MEG helmet to familiarize the children with the helmet and with padding their heads with a foam headband. Padding minimized head movements during recording and placed the small child head in a more centered position in the helmet.

Magnetoencephalography data were acquired during a no-task eyes-closed resting-state condition inside a magnetically shielded room (Vacuumschmelze GmbH, Hanau, Germany) using a 151-channel whole-head radial gradiometer MEG system (CTF Systems Inc., Port Coquitlam, BC, Canada) at the VU University Medical Center. A sample frequency of 625 Hz was used and the standard preprocessing steps undertaken at our lab to reduce noise are, spatial filtering using third order gradiometers (Vrba et al., 1999), band pass filtering, and visual inspection (see criteria in Data Processing) discarding data contaminated by artifacts. Visual inspection may be preferred over automatic approaches, since, for instance, an independent component analysis may introduce complex new artifacts or biases (Gross et al., 2013). Twelve sensors were not functioning during at least 1 of the recordings, and these were excluded from the analysis for all subjects. At the beginning and end of each recording, the head position relative to the coordinate system of the helmet was recorded by leading small alternating currents through three head position coils attached to the left and right preauricular points and the nasion. Head position changes during the recording of up to 1.5 cm were accepted, to which all children complied. The distance between the cortical mantle and the MEG sensors might have influenced the amplitude of the measured oscillatory signal. As an approximation of the distance of the cortical mantle to the MEG sensors, we calculated the distance of three coils (placed at the nasion, the left ear, and right ear preauricular fiducials) from the center of the MEG helmet.

During the resting-state condition, children were lying comfortably, and were instructed after 1 min to close their eyes for 4 min and move as little as possible. One of the investigators stood nearby the child to control for his/her well-being and one of the parents or caretakers was seated in a corner of the shielded room to comfort the child when necessary.

### Data processing

For off-line processing, the recordings were converted to ASCII format. Visual inspection and selection of the time segments was done by one of the investigators (MB) with BRAINWAVE software (CS, http://home.kpn.nl/stam7883/brainwave.html). For each child we selected five artifact-free time segments of 4096 samples (∼6.5 s) from the 4 min eyes-closed condition. This has proven to be sufficient to detect clinically relevant differences in power in previous MEG studies (De Haan et al., 2008; Bosboom et al., 2009) and stable over time within healthy subjects (Olde Dubbelink et al., 2013).

Typical artifacts were related to (eye) movement, drowsiness, or muscle contractions. One SGA+ child was excluded from further analysis due to continuous artifacts caused by construction activities near the scanner room. Three children showed minor abnormalities in their MEG recordings (two AGA, one SGA+). These patterns were characterized as spike and wave discharges and sharp waves. Besides these deviant patterns, the background MEG appeared normal in all of these children and these children had no history of epileptic seizures or other clinical symptoms. Since this deviant activity might affect the outcome, we excluded these three children from further analysis.

**Figure 1:**
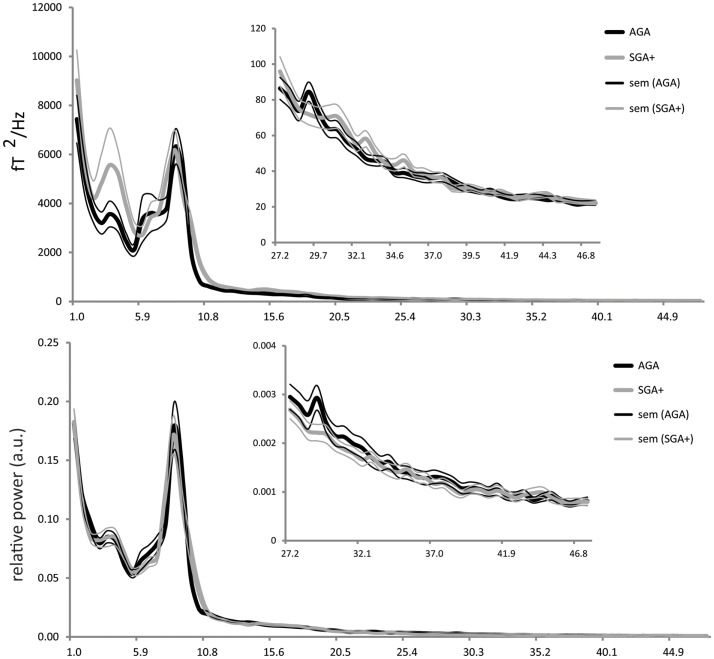
**Absolute power (*y*-axis: absolute power in fT2/Hz; *x*-axis: frequency in Hertz) and relative power (*y*-axis: arbitrary units; *x*-axis: frequency in Hertz) averaged for the AGA and SGA born groups, per group averaged over all MEG sensors (five epochs per child)**. Thick solid black lines represent the average of AGA born children; thick gray lines the SGA+ born children (standard errors of the mean are represented in thin lines).

For each selected time segment, we converted the MEG signal per sensor from the time domain into the frequency domain (Welch periodogram; matlab 7.0; window = 1024 samples; overlap = 512 samples; nfft = 1024; sample frequency = 625 Hz). Per sensor, the absolute power spectrum ranging from 1 to 48 Hz was divided into 78 bins of 0.618 Hz. For relative power spectra construction, the absolute power for each bin was divided by the absolute power of the total spectrum, resulting in values ranging between 0 and 1. It is well known that children at school age show a reduced alpha peak frequency (∼8 Hz) (Marshall et al., 2002; Boersma et al., 2011) compared to adults (∼10 Hz) (Cragg et al., 2011; Smit et al., 2011). Consequently, the alpha frequency would be split up at its peak in case standard definitions of adult lower and upper alpha frequency bands were to be used, which could introduce biases into the results. To ensure that all alpha-like frequencies are captured in children at school age, we chose to use the recorded profile (group averaged) power spectrum (shown in Figure 1), which shows clear theta and alpha peaks, to define new boundaries for the theta and alpha band that are appropriate for our population: total spectral power 1–48 Hz, delta 1.0–2.9 Hz, theta 2.9–5.4 Hz, alpha 5.4–11.5 Hz, beta 11.5–27.3 Hz, and gamma 27.3–48 Hz. Subsequently, per sensor, we computed the spectral power within these frequency bands by averaging over bins within these frequency ranges. Then, we averaged these outcomes over all MEG sensors (except 12 noisy or broken sensors), and subsequently, the outcomes were averaged over epochs (five per child) for each child.

To avoid possible biases of the selection of the frequency bands, additional analysis of absolute and relative power in 78 frequency bins was performed. We furthermore performed a region based analysis by grouping sensors in left and right anterior, left and right posterior and left and right temporal areas and subsequently averaged relative and absolute power over the sensors in each region for the different frequency bands.

### Estimation of intelligence and physical measurement

Both physical examination and estimations of intelligence were performed ranging from 36 weeks before to 23 weeks after MEG investigation. An estimate of the full scale IQ (eIQ) was based on a four-subtest short version of the much used and well standardized Wechsler’s scales that would ordinarily be obtained by administration of the complete scales. Estimates of reliability and validity indicate that the abbreviated forms of the Wechsler Preschool and Primary Scale Intelligence – Revised (WPPSI-R, Dutch version), for children under 6 years and the Wechsler Intelligence Scale for Children – third Edition (WISC-III, Dutch version) for children 6 years and older may be used to approximate the Full Scale IQ when time limitations are a consideration (Kaufman et al., 1996; LoBello, 1991). Parental educational levels were classified according to the International Standard Classification of Education (1997).

### Statistics

Statistical analyses were performed using SPSS 15.0 (Chicago, IL, USA). We used non-parametrical tests for all group comparisons because of the small group sizes and a skewed distribution of spectral power. To test for group differences in subject characteristics, Mann–Whitney *U*-tests were used for continuous data (eIQ, body weight, length, head circumference) and Fisher exact tests were used for categorical variables (gender, handedness, parental education). Mann–Whitney *U*-tests were used to test for differences between AGA and SGA groups in absolute and relative power in several frequency bands. *Post hoc* analyses were done to investigate the relationships between total absolute power and continuous variables, which included IQ, head circumference, and the distance between head position coils and the MEG helmet, by using Spearman’s rank correlations. For all analyses, *p*-values < 0.05 (two-tailed) and *Z* values (ignoring the sign) greater than 1.96 were considered statistically significant.

Additional analysis using log-transformed data and *t*-tests for independent samples for absolute and relative power for 78 frequency bins were performed to avoid possible biases of the selection of the frequency bands. To statistically test for group differences in region based analysis, we used log-transformed data, *t*-tests for independent samples and FDR correction for multiple testing.

## Results

### Subject characteristics

Table 1 lists subject characteristics per group and the significance of the differences between groups. Distributions of handedness and gender did not differ between groups. Twenty-one children were right-handed and the two left-handed children were equally distributed over the subgroups. Groups did not differ in gestational age. Conform the definition, children born SGA had a significantly lower birth weight than AGA born children. Furthermore, although relatively spared compared to body weight and length, head circumference at birth was significantly lower in SGA compared to AGA children. Age at MEG recording did not differ between the groups. Body length at the MEG recording was significantly lower in SGA children compared to AGA. Although catch-up growth had occurred in all SGA+ children, mean length SD was just below population mean. In contrast, complete catch-up of head circumference (mean SD = −0.08) had occurred. Nevertheless, head circumference in SGA+ was significantly lower compared to AGA children. This difference is possibly due to AGA children having a head circumference slightly above average compared to the Dutch population mean, whereas SGA+ children showed head circumference comparable to the Dutch population mean. For body length, all SGA+ children stayed within −2 SD of the mean according to definition. A trend toward significance was found with higher eIQ in AGA children compared to SGA+ children. As the control group shows an IQ of 120, the IQ difference (at trend level, *p* = 0.058) between groups indicates that the control group shows intelligence above average instead of reduced intelligence in SGA children. The proportion of parents in the SGA group with an educational level confined to first stage of basic education or lower secondary education did not differ from the parents in the AGA group (3 mothers SGA+ vs. 0 mothers AGA, Fisher exact *p* = 0.853; 3 fathers vs. 0 fathers AGA, Fisher exact *p* = 0.443).

**Table 1:** **Subject characteristics**.

	AGA (*n* = 10)	SGA+ (*n* = 13)	AGA vs. SGA+		
	Mean ± SD	Mean ± SD	*U*	*Z*	*p*
Gender (boy: girl)	7:3	6:7			0.428^a^
Handedness (right: left)	9:1	12:1			0.999^a^
Gestational age (weeks)	39.4 ± 1.2	38.4 ± 2.0	46.5	−1.151	0.25
Weight at birth (grams)	3464 ± 489	2144 ± 407	1.0	−3.840	**<0.001**
Weight at birth compared to Dutch population (SD^reference^)	0.10 ± 0.88	−2.61 ± 0.40	0.0	−4.037	**<0.001**
Head circumference at birth compared to Dutch population (SD^reference^)	0.21 ± 0.67	−1.09 ± 0.75	8.0	−3.434	**<0.001**
Age at MEG investigation (years)	5.97 ± 1.2	6.28 ± 0.87	55.5	−0.589	0.556
IQ estimated	114.2 ± 12.2	104.8 ± 11.8	34.5	−1.897	0.058
Length at MEG investigation (cm)	118.1 ± 10.5	111.6 ± 7	44.5	−1.272	0.203
Length at MEG investigation compared to Dutch population (SD^reference^)	0.22 ± 1.02	−0.59 ± 0.75	31.0	−2.110	**0.035**
Weight at MEG investigation (kg)	23.10 ± 8.10	17.2 ± 6.2	28.0	−2.112	**0.035**
Weight at MEG investigation compared to Dutch population (SD^reference#^)	−0.05 ± 0.85	−0.67 ± 1.08	36.0	−1.800	0.072
Head circumference at MEG investigation (cm)	52.0 ± 1.30	51.0 ± 1.2	29.5	−1.749	0.080
Head circumference at MEG investigation compared to Dutch population (SD^reference^)	0.59 ± 0.68	−0.08 ± 0.65	26.5	−2.398	**0.016**

^a^ Fisher exact; Body weight and length, as well as head circumference, are expressed in both absolute measures as well as in deviations from the Dutch population mean (where the deviation is expressed as fraction of the SD of the reference population, SD^reference^); ^#^SD according to length. Data (except gender and handedness) are presented as mean (±SD). Bold text indicates a statistical significant difference (p < 0.05).

### Power spectra

As shown in Figure 1, the absolute power spectra for SGA born children and AGA born children largely overlap. Overall, the SGA group has comparable absolute power over the whole frequency range. More specifically, group comparison showed that AGA and SGA+ groups did not differ significantly in absolute power in all frequency bands except for the gamma band, in which SGA+ had lower gamma power (for statistics see Table 2).

**Table 2:** **Test statistics for absolute and relative power**.

	AGA vs. SGA+		
	*U*	*Z*	*p*
**ABSOLUTE POWER**			
Total	46.000	−1.18	0.239
Delta	45.000	−1.24	0.215
Theta	51.000	−0.87	0.385
Alpha	43.000	−1.36	0.172
Beta	50.000	−0.93	0.352
Gamma	27.000	−2.36	**0.018**
**RELATIVE POWER**			
Delta	61.000	−0.25	0.804
Theta	51.000	−0.87	0.385
Alpha	52.000	−0.81	0.420
Beta	61.000	−0.25	0.804
Gamma	58.000	−0.43	0.664

Bold text indicates a statistical significant difference (p < 0.05).

Figure 1 showed that the relative power spectra for the SGA+ and AGA group largely overlap. Relative power did not differ between groups for the total spectrum, nor for the separate frequency bands (for statistics see Table 2). In summary, the SGA group shows similar frequency distributions of the brain oscillations, with similar amplitudes except for the gamma band.

When performing additional analysis testing for group differences in 78 frequency bins, we did not find significant group differences in both absolute and relative power in any of the 78 frequency bins, even when no correction for multiple testing was performed.

We did not find significant group differences when evaluating regional functional differences in left and right anterior, posterior and temporal regions.

### *Post hoc* analyses

We tested whether groups differed in distances between the head and the MEG helmet as this might influence the amplitude of the measured oscillatory signal. AGA and SGA did not show significantly different distances (*U* = 95.0, Z = −0.310, *p* = 0.757). We found significant smaller head circumferences (in SD) in SGA children compared to AGA children (Table 1), though we did not find a relation between head circumference and total spectral power in both AGA and the SGA group. Furthermore, we did not find significant correlations between eIQ and absolute or relative power in both groups.

## Discussion

In this study, we tested if oscillatory brain activity is affected in term born SGA born children at early school age, as suggested by the findings of a previous EEG study. Only SGA born children in which postnatal catch-up growth had occurred were included. SGA+ children showed significantly lower absolute power than AGA children in the gamma band, but not in other frequency bands. The relative power spectra did not significantly differ between AGA and SGA groups. A trend toward significance with higher eIQ in AGA children compared to SGA children was found. Estimated IQ did not correlate with absolute or relative power in both groups. Head circumference differed between AGA and SGA children however HC was not correlating with absolute power in both groups.

In contrast to the EEG-based results by Ozdemir et al. (2009), we found that relative and absolute power did not differ between SGA born children at early school age compared to AGA children, except for lower absolute gamma band power in SGA born children. This frequency band was not examined in the neonates investigated by Ozdemir et al. Furthermore, distinguishing true brain gamma band oscillations from gamma band activity caused by muscle artifacts using EEG is difficult as recently has been shown (Whitham et al., 2007). Unlike EEG, MEG has the advantage of being insensitive to skull-thickness and skin-conductance (Ent et al., 2009), and no reference electrode is required. Possible differences in skull-thickness between AGA and SGA born children, could therefore not have affected our estimated MEG power spectra. On the other hand, MEG might be more influenced by head movements and might favor tangential sources [but see (Hillebrand and Barnes, 2003)]. Although in the present study visual inspection of the lower frequency ranges in the absolute power spectrum might suggest group differences, additional statistical tests based on log-transformed data did not show significant differences between groups in the delta and theta bands. Furthermore, supplemental analyses for log-transformed data did not show significant group differences in both absolute and relative power in any of the 78 frequency bins. Moreover, groups did not significantly differ when evaluating regional functional differences in left and right anterior, posterior and temporal regions.

Few other studies investigated the effect of (extreme) low birth weight on spectral power in neonates and adults (Grieve et al., 2008; Miskovic et al., 2009), however, these studies included preterm births. Distinction between the effects of IUGR and the effect of gestational age and prematurity is difficult since prematurity also affects oscillatory activity as shown in a recent MEG study (Doesburg et al., 2011). They found reduced alpha band power and slowing of the peak frequency in 7-year-old children who were born very premature. Young adults born preterm with a very low birth weight (VLBW) showed significantly higher relative spectral power in the lower frequency bands and lower relative power in higher frequency bands compared to adults born with normal birth weight (NBW) (Miskovic et al., 2009) suggesting a maturational delay of the brain that persists into adulthood. Different from these findings, our groups showed highly overlapping absolute and relative power spectra demonstrating that at early school age the ratios of low and high frequency oscillations are comparable between term born SGA+ and AGA born children. This suggests that the development of brain oscillatory activity is not delayed at early school age in SGA born children born at term.

Strong associations have been described in the literature between IQ and oscillatory activity in resting-state conditions, implicating that the basic resting-state condition is highly informative about a brain’s cognitive capabilities. Thatcher et al. (2005) reported positive correlations between IQ and absolute power for all frequency bands in a group of healthy subjects with an age range from 5 to 52 years old. Schmid et al. (2002) found a positive relationship between IQ and relative alpha band power, and a negative correlation between IQ and lower frequency band relative power in school age children. We did not find significant correlations between eIQ and absolute or relative power for both groups. The mean IQ’s in our sample are relatively high which may not appear to be typical to SGA once we study larger samples. Future research in larger groups should further investigate the effects of being born SGA and catch-up growth on the association between intelligence and brain oscillatory activity.

This study was limited by a few factors. It has previously been reported that EEG abnormalities of mild nature occur more often in SGA newborns than in a control group (Fitzhardinge and Steven, 1972). One SGA and two AGA born children exhibited (occasional) spikes and/or spike and wave patterns without apparent clinical symptoms. Since deviant activity might have influenced the outcome, we decided to exclude these children from further analysis. Unfortunately, we were not able to enlarge the sample size due to scanner replacement. Furthermore, we only included SGA+ children. Future studies should test for the effect of bodily catch-up growth on oscillatory brain activity in SGA children.

What might be the biological mechanisms that underlie the reduction in absolute gamma power observed in SGA born children? The oscillatory activity measured by MEG is assumed to originate from large networks of spatially and temporally organized neurons and is dependent on local synaptic density and cortical volume, which is shown to be lowered as smaller volumes are found in SGA born children (de Bie et al., 2011). The amplitude of the signal also depends on the synchronizability of the underlying networks, which in turn depends on local connectivity patterns between the excitatory and inhibitory neurons within these networks. In the present study changes in neural mass/local neural circuits might have only led to reductions in the amplitude (absolute power) without changing the frequency of the oscillations (which would have been reflected in the relative power). Especially gamma band activity has previously been associated with perception and attention and cortical local short-distance connections are thought to underlie the high frequency oscillations (Jensen et al., 2007). During pre- and postnatal maturation of the brain, the ability of neurons to migrate, differentiate, and connect to other neurons is important for the formation of optimally organized networks and for the establishment of synchronous activity in these networks. The timing and severity of growth restriction *in utero* determines which brain maturation processes are affected and what the impact of intra-uterine growth restriction will be later in live (Kostovic and Jovanov-Milosevic, 2006; Kostovic and Judas, 2006; Rees et al., 2008). Disturbances of these processes early in life might have affected the ability to synchronize these networks. Future studies should further investigate if severity of IUGR early in life is related to gamma power and for instance attention and behavior later in life.

In conclusion, the great overlap in both relative and absolute power suggests normal development of brain oscillatory activity at early school age in term born SGA children when spontaneous bodily catch-up growth has occurred. An important question that remains to be answered, is how disturbances early in development affect the organization of functional brain networks and its dynamics when SGA born children do not show spontaneous bodily catch-up growth.

## Conflict of Interest Statement

The authors declare that the research was conducted in the absence of any commercial or financial relationships that could be construed as a potential conflict of interest.
